# A 6-tsRNA signature for early detection, treatment response monitoring, and prognosis prediction in diffuse large B cell lymphoma

**DOI:** 10.1038/s41408-025-01267-z

**Published:** 2025-04-28

**Authors:** Jun Rao, Lin Xia, Qiong Li, NaYa Ma, Xinlei Li, Jiali Li, Lidan Zhu, Pan Zhao, Yunjing Zeng, Sha Zhou, Huanping Guo, Shijia Lin, Song Dong, Shifeng Lou, Fangyi Fan, Jin Wei, Jiang F. Zhong, Li Gao, Shengwen Calvin Li, Xi Zhang

**Affiliations:** 1 Medical Center of Hematology, Xinqiao Hospital, Army Medical University, Chongqing, China; 2 State Key Laboratory of Trauma and Chemical Poisoning, Chongqing Key Laboratory of Hematology and Microenvironment, Chongqing, China; 3 National Clinical Research Center for Hematologic Diseases, The First Affiliated Hospital of Soochow University, Soochow, China; 4 Department of Hematology, The Second Affiliated Hospital of Chongqing Medical University, Chongqing, China; 5 Department of Hematology, General Hospital of Chengdu Military Region, Chengdu, Chongqing, China; 6 Department of Hematology, North Sichuan Medical College, Nanchong, China; 7 Department of Medicine, Keck School of Medicine, University of Southern California, Los Angeles, California, CA USA; 8 CHOC Children’s Research Institute, Children’s Hospital of Orange County (CHOC®), part of Rady Children’s Heath, Orange, CA USA; 9 Department of Neurology, University of California-Irvine School of Medicine, Orange, CA USA; 10 Jinfeng Laboratory, Chongqing, China

**Keywords:** Cancer genetics, Genetics research

## Abstract

Diffuse large B-cell lymphoma (DLBCL) presents considerable clinical challenges due to its aggressive nature and diverse clinical progression. New molecular biomarkers are urgently needed for outcome prediction. We analyzed blood samples from DLBCL patients and healthy individuals using short, non-coding RNA sequencing. A classifier based on six tsRNAs was developed through random forest and primary component analysis. This classifier, established using Cox proportional hazards modeling with repeated 10-fold cross-validation on an internal cohort of 100 samples analyzed using RT-qPCR, effectively identified high-risk patients with significantly lower overall survival compared to low-risk patients (Hazard ratio: 6.657, 95%CI 2.827-15.68, *P* = 0.0006). Validation in an external cohort of 160 samples using RT-qPCR confirmed the classifier’s robust performance. High-risk status was strongly associated with disease histological subtype, stage, and International Prognostic Index scores. Integration of the classifier into the IPI model enhanced the precision and consistency of prognostic predictions. A dynamic study revealed that patients experiencing a 1.06-fold decrease after one therapy cycle (early molecular response) exhibited better treatment outcomes and prognosis. Furthermore, the 6-tsRNA signature accurately differentiated healthy individuals from DLBCL (AUC 0.882, 95%CI 0.826-0.939). These findings underscore the potential of the identified 6-tsRNA profile as a biomarker for monitoring treatment effectiveness and predicting DLBCL outcomes.

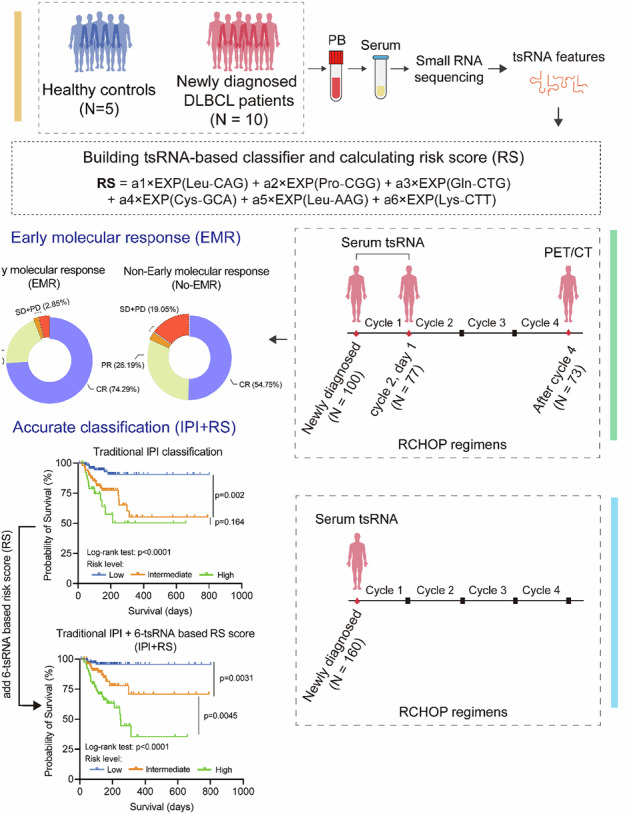

## Introduction

Diffuse large B cell lymphoma (DLBCL) is a highly aggressive and heterogeneous non-Hodgkin lymphoma [1], constituting up to 40% incurable with hypermutations of oncogenes in all cases globally [2, 3]. Due to its notable clinical heterogeneity and evolving tumor microenvironment, 30–40% of patients are unresponsive or relapse, thereafter making prognostication and therapeutic decisions challenging consequently [4–6]. To address the heterogeneity using gene expression profiling of cell-of-origin (COO), DLBCL is distinguished into germinal center B cell-like (GCB), activated B cell-like (ABC), and unclassified subtypes [7].

Recently, significant progress has been made in enhancing comprehension of the molecular characteristics of DLBCL, leading to the successful classification of the original 3 subtypes into more refined categories [8, 9]. Upon such refined classification, DLBCL patients with specific subgroups benefited from combined rituximab and chemotherapy and achieved higher response rates [10]. However, 30–40% of patients would develop resistance, and the responses are short-lived with a widely recognized standard regimen, R-CHOP (rituximab plus cyclophosphamide, doxorubicin, vincristine, and prednisone) [11–14]. Moreover, not only treatment-driven molecular features-based diagnostics require a series of spatiotemporal tumor biopsy analyses, but also the invasive procedures presented only a snapshot of DLBCL heterogeneity, which was inconvenient and unfeasible during long-term follow-up to determine subclonal evolution [15], particularly for high-grade B-cell lymphoma with translocations involving MYC and BCL2 or BCL6, usually referred to as double-hit lymphoma (DHL) [16]., non-invasive biomarkers are urgently required to develop and monitor DLBCL treatment response and disease progression.

Liquid biopsy is an effective and non-invasive procedure that detects molecular characteristics of tumors in peripheral blood, including circulating tumor DNA [cell-free fragments of DNA (cfDNA)] or circulating-free coding and non-coding RNA [17–19]. Emerging evidence has shown that circulating-free coding and non-coding RNA, including messenger RNA (mRNA), microRNA (miRNA) [20], long non-coding RNA (lncRNA), tRNA-related RNA fragments (tRFs) (AS-tDR-008946, AS-tDR-013492) [21], circular RNA (circRNA), RNA encapsulated in extracellular vesicles, extracellular circulating small non-coding RNA (sncRNA), function as promising biomarkers for DLBCL [22]. The tRNA-derived small non-coding RNA (tsRNA) is a class of newly identified small non-coding RNA (sncRNA), usually ~30 nucleotides (nt) in length, and can be divided into four major distinct categories: 5′tsRNA, inner’tsRNA, 3′tsRNA, and 3’CCA tsRNA, which are derived from 5′, internal, 3′, and 3′CCA of precursor tRNA or mature tRNA, respectively [23]. tsRNAs involved in cell proliferation, mRNA silencing, translation regulation, apoptosis inhibition, cell–cell communication, epigenetic regulation, and tumor metastasis via regulating ribosome biogenesis [24, 25], protein synthesis [26, 27], miRNA-like gene regulation [28–30], and epigenetic regulation [31, 32]. tsRNA is highly enriched in vertebrate serum, and serum tsRNAs manifest as diagnostic biomarkers for human diseases that occur upon starvation, oxidative stress, hypoxia, and other adverse conditions [33–36]. Dysregulation of circulating tsRNA and the specific signatures could serve as prognostic biomarkers and therapeutic targets in multiple cancers [37–40]. We recently found that non-classical sncRNAs, including rsRNA (62.86%), ysRNA (14.97%), and tsRNA (4.22%), dominated among serum sncRNAs and showed alteration patterns closely associated with acute myeloid leukemia (AML) prognosis [39]. However, the expression features and diagnostic values of serum tsRNA for DLBCL are still ambiguous and intriguing.

Here, by employing sncRNA sequencing and RT-qPCR on the serum of DLBCL patients, we presented the landscape of tsRNA profiles in patients, identified significantly dysregulated tsRNA, and developed a 6-tsRNA-based classifier for early detection, drug response, and prognosis of DLBCL.

## Materials and methods

### Study design and sample collection

In this retrospective cohort study, three cohorts were incorporated. Serum samples were prospectively collected at newly diagnosed and after therapy if possible, namely (1) *the discovery cohort*: 15 serum samples in total, including 5 healthy individuals and 10 newly diagnosed DLBCL patients enrolled in the hematology medical center of Xinqiao Hospital from February 2020 to December 2021; (2) *the internal validation cohort*: 135 serum samples in total, including 35 healthy individuals and 100 newly diagnosed DLBCL patients enrolled in the hematology medical center of Xinqiao Hospital from February 2020 to December 2021. Among the 100 newly diagnosed patients and 77 patients’ serum samples were collected at cycle 2, day 1; (3) *the external validation cohort*: 160 serum samples in total, and all samples are newly diagnosed DLBCL patients enrolled at four centers across China between October 2020 and August 2022. For all three cohorts’ demographic details, please see Supplemental Table 1.

All consecutive DLBCL patients who were deemed appropriate for this study during the study period were included without selection. Histological diagnoses were established independently by at least two experienced senior pathologists according to the WHO classification of Tumors of Hematopoietic and Lymphoid tissue criteria. Upon diagnosis, all patients underwent baseline staging using laboratory, radiographic, and bone marrow examinations. Eastern Cooperative Oncology Group (ECOG) performance status was also assessed. The stage was evaluated following the Ann Arbor staging system. The International Prognostic Index (IPI) was calculated based on serum lactate dehydrogenase, stage, extranodal status, and performance status. Patient characteristics and treatment regimens of each therapy cycle were collected. Healthy individuals were recruited as control participants for small non-coding RNA sequencing and RT-qPCR. After the initial stage assessment, all DLBCL patients were given 6–8 cycles of RCHOP regimens (750 mg/m^2^ cyclophosphamide, 50 mg/m^2^ doxorubicin, and 4 mg of vincristine (all day 1), and 60 mg/m^2^ prednisone on days 1–5, with 375 mg/m^2^ rituximab on day 0) as the previous report [37]. Patients were reviewed routinely by a combination of clinical assessment and CT or fluorodeoxyglucose-PET (FDG-PET) before the administration of chemotherapy. FDG-PET was planned as an interim scan.

### Peripheral blood serum isolation

Serum isolation of peripheral blood samples was performed as previously described [39]. Peripheral blood samples were collected using 5 ml non-anticoagulative vacuum blood collection tubes (Cat#ZL 5 mL, 20132411377, JinXing, China). To promote clotting, peripheral blood samples were placed at room temperature for 30 min. After peripheral blood coagulation, the serum was separated by centrifuging at 3500×*g* at 4 °C for 15 min, and then isolated serum was transferred into new tubes. Subsequently, to thoroughly remove cell debris contamination, isolated serums were centrifuged again at 12,000×*g* for 10 min, and then isolated serums were transferred into new tubes. Lastly, all the samples were stored at −80 °C for further RNA extraction.

### Serum RNA extraction

Serum RNA was extracted using TRIzol LS reagent (Cat#10296028, Invitrogen, USA) according to the manufacturer’s protocol and performed as previously described [39]. Briefly, 0.25 ml serum was added to a 1.5 ml tube mixed with 0.75 ml TRIzol LS reagent and swirled. The mixtures were incubated on ice for 2 h with occasional vortexing to ensure the serum was completely cracked. Then, 0.2 ml chloroform was added to the mixtures, vortexed, and incubated at room temperature for 10 min. The samples were centrifuged at 12,000×*g* for 15 min at 4 °C. Next, the aqueous phase was collected into a tube filled with an equal volume of isopropanol. Then, the mixtures were gently mixed with 1.5 μl glycogen (Cat#10901393001, Roche, Switzerland) and refrigerated at −80 °C for at least 30 min to precipitate RNA. The mixture was centrifuged at 12,000×*g* for 30 min at 4 °C, and the pellet was washed with 75% cold ethanol twice. Finally, the RNA pellet was dissolved in 10 μl of RNase-free water after being dried and stored at −80 °C for RT-qPCR.

### Reverse transcription and quantitative real-time PCR (RT-qPCR)

To measure tsRNA expression levels in each sample, reverse transcription for validation was performed as previously described [41]. Firstly, the tsRNA was reverse-transcribed. Briefly, total serum RNA from each object was polyadenylated using *E. coli* Poly (A) polymerase (Cat#0276 L, NEB, USA) and then converted to cDNA in M-MuLV reverse transcriptase reaction system (Cat#M0253L, NEB, USA) with a unique adaptor (3′universal primer, supplemental Table 2). Then, tsRNAs were amplified from cDNA using specific tsRNA primers (5′ primer for tsRNA, Supplemental Table 2) in combination with the universal adaptor in a 10 μl reaction system containing 5 μl of TB Green® Premix Mix (Cat#RR820A, Takara, Japan), 1 μl (700 ng) of cDNA template, 0.25 μl (10 μM) of each primer, and 3.5 μl of distilled water. The cycling conditions were conducted following GoTaq® Green Master Mix instructions: step1, 95 °C for 5 min; step2, 95 °C for 30 s; step3, 60 °C for 30 s; step3, Melt Curve Stage; step2 to step3 for 40 cycles), and analyzed by a CFX Connect Real-Time PCR Detection System (Bio-Rad, Hercules, CA, USA). In addition, U6 was used as an internal control for normalization between samples (5′ and 3′ primer for U6, Supplemental Table 2).

### Small RNA library construction, sequencing, and small RNA-seq analysis

Small RNA library construction and sequencing were performed by BGI (Shenzhen, Guangdong, China) according to the NEB Small RNA Sample Pre Kit (NEB). Firstly, total RNA was extracted from the serum using TRIzol LS reagent (Invitrogen, Carlsbad, CA, USA), and then Agilent 2100 Bioanalyzer (Agilent, Santa Clara, USA) was used to test sample integrity and concentration, and NanoDrop (NanoDrop, Madison, DC, USA) to Inorganic ions or polycarbonate contamination. Subsequently, total RNA (200 ng–1 µg) was separated by 15% urea denaturing polyacrylamide gel electrophoresis (PAGE), and small RNA regions corresponding to the 15–50 nt bands in the marker lane were excised and recovered. Then, the small RNAs were firstly ligated to adenylated 3′ adapters (NEB Small RNA Sample Pre Kit); (Reaction condition: 70 °C for 2 min; 25 °C for 1 h), secondly add RT-Primer, (Reaction condition: 75 °C for 5 min; 37 °C for 15 min; 15 °C for 25 min) and thirdly add 5′adaptor mix system (Reaction condition: 70 °C for 2 min; 25 °C for 1 h); The adapter-ligated small RNAs were subsequently transcribed into cDNA by Superscript II Reverse Transcriptase (Invitrogen, Carlsbad, CA, USA) (Reaction condition: 70 °C for 2 min; 50 °C for 1 h) and then several rounds of PCR amplification with PCR Primer Cocktail and PCR Mix were performed to enrich the cDNA fragments (Reaction condition: 94 °C for 30 s; 11–13 cycles of (94 °C for 15 s, 62 °C for 30 s, 70 °C for 15 s); 70 °C for 5 min; 4 °C hold). Then, the PCR products were purified with PAGE gel. Dissolve the recycled products in EB solution. The library was qualitative and quantitative in two ways: the size distribution of the fragments was verified using the Agilent 2100 bioanalyzer, and the library was quantified by real-time quantitative PCR (QPCR) (TaqMan Probe). The final ligation PCR products were sequenced using the Illumina-HiSeq 2500 platform.

Raw sequencing reads were processed using the software SPORTS (version 1.1) [42]. Firstly, the data was cleaned by removing 5′ and 3′ adapters and discarding low-quality reads; only 15 nt–45 nt insertions were kept for further analysis. Then, clean reads were further aligned to the human reference genome (GRCH38/hg38, UCSC, https://genome.ucsc.edu) and small non-coding RNA relative databases, which include miRNA datasets (miRbase, version 21, source: http://www.mirbase.org/index.shtml), rRNA and YRNA datasets (NCBI nucleotide and gene database, source: https://www.ncbi.nlm.nih.gov/nuccore) (Hizir et al., 2017), genomic tRNA datasets (GtRNAdb, version 71, source: http://gtrnadb.ucsc.edu/), mitochondrial tRNA datasets (mitotRNAdb, version 72, source: http://mttrna.bioinf.uni-leipzig.de/mtDataOutput/), piRNA datasets (piRBase, version 29, source: http://www.regulatoryrna.org/database/piRNA/; piRNABank, version 30, source: http://pirnabank.ibab.ac.in/index.shtml), non-coding RNA datasets (Ensembl, release 89, source: http://www.ensembl.org/index.html; Rfam, version 12.3, source: http://rfam.xfam.org/).

Subsequently, tsRNAs were divided into four major distinct categories: 5’tsRNA, inner’tsRNA, 3′tsRNA, and 3′CCA tsRNA, which are derived from 5′, internal, 3′, and 3’CCA of precursor tRNA or mature tRNA, respectively. Finally, the sncRNA expression level was normalized to the total count of the reads that matched the genome of each sample separately (RPM: Reads of exon model per Million mapped reads). Note that there is no consensus or uniform standard for the reference gene in RT-qPCR analysis of serum tsRNAs. U6 and cel-mir-39 are the most commonly used control genes in RT-qPCR assays of serum small RNA. Additionally, we tested the feasibility of using cel-mir-39 in some of the DLBCL samples, and the results were consistent.

### 6-tsRNA-based prognosis prediction

The 6-tsRNA selection was performed based on the following pipeline. Firstly, based on the tRNA reference we used, 37 differentially expressed tsRNA were kept for further analysis (the absolute value of Log2FC > 1 and *p* < 0.05). Subsequently, the top 10 tsRNAs that contribute significantly to the accuracy of classification were kept (based on the importance of features by employing random forest and PCA). Finally, we accounted for the following: (1) certain tsRNA sequences exhibit high homology and can be considered as a single tsRNA; (2) primer efficiency is crucial for accurate tsRNA detection via RT-qPCR, so tsRNAs that produced non-specific bands have been excluded from the analysis. As a result, six tsRNA, including tsRNA-Leu-CAG, tsRNA-Pro-CGG, tsRNA-Gln-CTG, tsRNA-Cys-GCA, tsRNA-Leu-AAG, and tsRNA-Lys-CTT, were selected for further analysis (the mapping information on the single-base resolution of their precursor tRNAs was presented in supplemental Fig. 1 and Supplemental Table 2).

The 6-tsRNA-based prognosis prediction model was performed on the internal validation cohort (newly diagnosed DLBCL patients (*n* = 100) were included in the following analyses, and 1000-times repeated 10-fold cross-validation was performed. Briefly, with each iteration of the Cox proportional hazards model, one set of samples (*n* = 10) was randomly sampled and left out first. The remaining samples (*n* = 90) were used as a training dataset for tsRNA modeling. Subsequently, the left-out samples were used as an independent testing dataset to evaluate the prediction accuracy of tsRNA models. In particular, 1000 LOO iterations were set and performed in the Cox proportional hazards model, and the averaged coefficient was used. The tsRNA feature section was performed using random forest (randomForest, version 4.7-1.1) and PCA (factoextra, version 1.0.7; FactoMineR, version 2.9) methods. 6-tsRNA-based prognosis prediction and the accuracy of tsRNA models were calculated by the concordance index (c-index) (survival, version 3.5–7; rms, version 6.7-1).

### Statistical analysis

Statistical analysis was conducted using SPSS software (Version 18.0, LEAD Corp). Descriptive statistics were used to analyze the characteristics of clinical, demographic, and genetic test results. The correlation of tsRNA levels and clinicopathologic features of patients was conducted using the Pearson *χ*2 tests. The overall survival (OS) is measured as time from initiation of induction treatment to death resulting from any cause. The progression-free survival (PFS) was defined as the time duration from diagnosis until relapse or progression, unplanned re-treatment of lymphoma after initial immunochemotherapy, or death as a result of any cause. Survival estimates were obtained using the Kaplan–Meier method, and comparisons were made using a log-rank test. Unpaired Student’s *t*-test for two groups was used in this study. The prediction potential of the risk model was used in the timeROC package in R, and the AUC (area under the curve) value measures the overall performance of a binary classification model and provides an intuitive way to compare different models. COX proportional regression model was used to calculate the survival hazard ratio (HR). Statistical differences were considered significant if the *P* value was less than 0.05.

### Ethics approval and consent to participate

All experiments were performed in accordance with the principles outlined in the World Medical Association Declaration of Helsinki. This study was approved by the Institutional Ethics Committees of Xinqiao Hospital, and informed consent was signed by each participant.

### Data sharing statement

The sncRNA sequencing dataset generated during the current study (including serum samples of 10 DLBCL patients and five healthy individuals) has been deposited into Genome Sequence Archive for Human (GSA-Human, https://bigd.big.ac.cn/gsa-human/) with accession number HRA002579.

## Results

### Clinical characteristics of DLBCL patients in all cohorts

To further delve into the tsRNA profiles, patient selection was consecutive and unbiased, with diagnoses established by experienced pathologists based on WHO classification criteria. In the whole cohort, the median age at the first blood draw in the validation cohort was 53 years (range, 18–81 years), and 160 patients were men. Based on the classification system, 128 patients were classified as GCB subtypes and 132 as Non-GCB subtypes. According to the Ann Arbor staging criteria, 42 patients were identified as stage I, 77 as stage II, 74 as stage III, and 67 as stage IV. The median follow-up duration of the validation cohort was 243.5 days (range, 48–801 days) (Table 1).

**Table 1:** Demographic and clinical characteristics of DLBCL patients.

	Healthy individuals		DLBCL patient		
Prognostic variables	No.	No.	Discovery cohort (*n* = 10,%)	Internal validation cohort (*n* = 100, %)	External validation cohort (*n* = 160, %)
*Gender*					
Male	21	165	5.00	63.00	60.63
Female	14	105	5.00	37.00	39.38
* Age (Years)*					
<53	16	119	7.00	47.00	40.63
≥53	19	151	3.00	53.00	59.38
*Histological Subgroup*					
GCB		132	5.00	37.00	56.25
Non-GCB		138	5.00	63.00	43.75
*Clinical stage*					
I		45	3.00	15.00	16.88
II		77	0.00	18.00	36.88
III		77	3.00	28.00	28.75
IV		71	4.00	39.00	17.50
*Final recurrence status*					
with		32	1.00	13.00	11.25
without		238	9.00	87.00	88.75
*B symptoms*					
with		47	2.00	19.00	16.25
without		223	8.00	81.00	83.75
*IPI Scores*					
0		24	1.00	21.00	1.25
1		42	4.00	24.00	8.75
2		82	3.00	14.00	40.63
3		73	2.00	27.00	27.50
4		49	0.00	14.00	21.88

### 6-tsRNA signatures serve as promising biomarkers for prognosis prediction

**Fig. 1:**
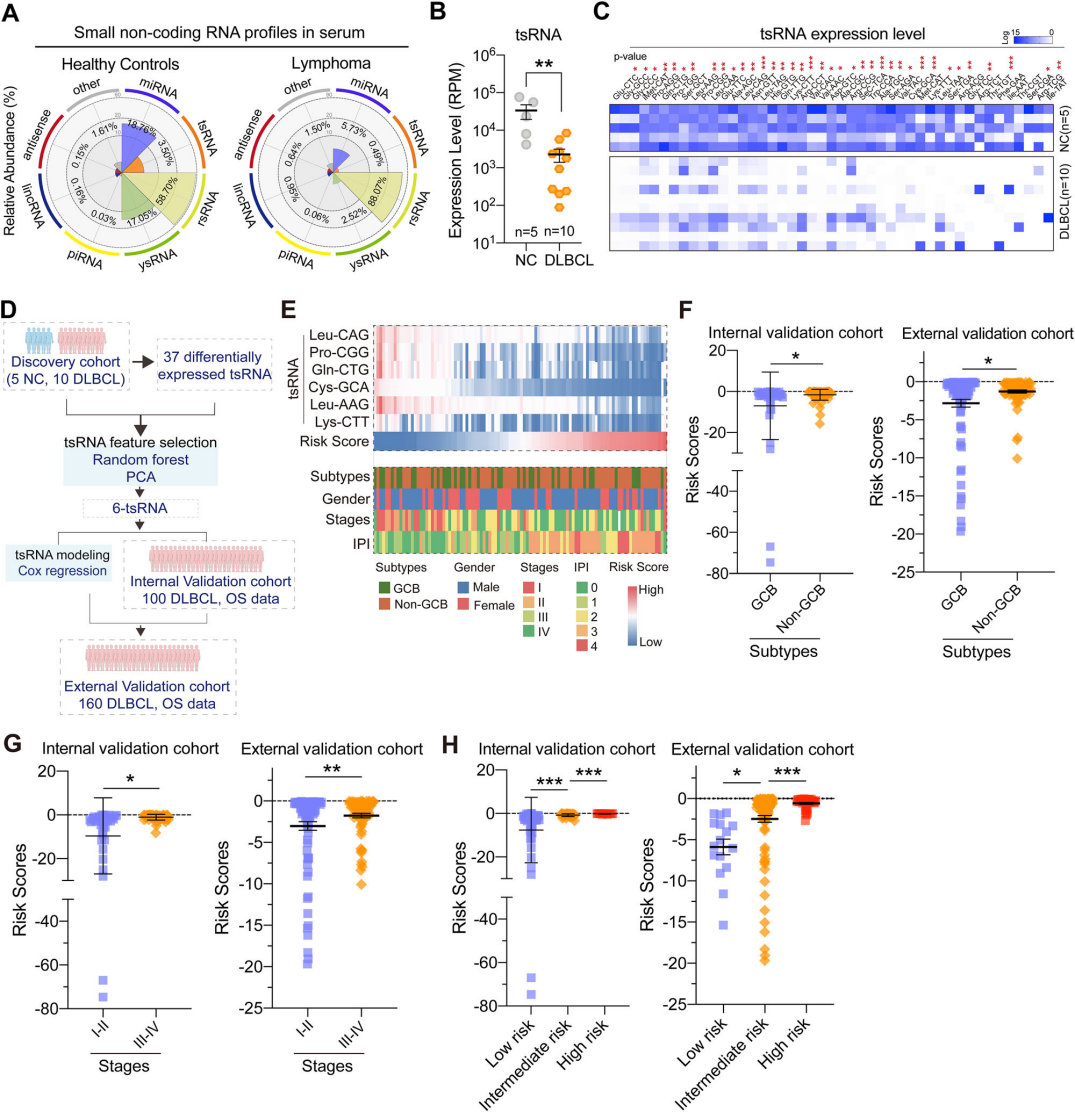
Identification of tsRNA signatures in serum of DLBCL patients. **A** The relative proportion of eight major sncRNA categories in DLBCL patients and healthy controls. **B** The relative expression level of tsRNA between DLBCL patients and healthy individuals, the expression level was presented as means ± SEM. **C** The heat map indicates the differences in tsRNA expression profiling between DLBCL patients and healthy individuals, and six tsRNA samples selected in (**D**) were highlighted. **D** Schematic overview of tsRNA feature selection. **E** The association between the clinical characteristics of DLBCL patients and individual tsRNAs, 6-tsRNA classifiers, and risk scores (RS) in the internal validation cohort (*n* = 100), with each column representing a sample. **F** Comparison of the distribution of risk scores between DLBCL histological subtypes in the internal validation cohort (*n* = 100) and external validation cohort (*n* = 160). **G** Comparison of RS in disease stages of DLBCL patients. **H** Comparison of the distribution of RS in risk group-based IPI scoring systems in DLBCL patients.

By scanning the small non-coding RNA sequencing in the discovery cohort, we found that the expression pattern of sncRNA was distinct between healthy individuals and DLBCL patients, in which tsRNA has changed significantly, suggesting that tsRNA might be more sensitive biomarkers for lymphoma detection (Fig. 1A, B). Specifically, compared with healthy individuals, the overall expression level of tsRNA was significantly decreased in DLBCL patients, and 37 tsRNAs were differentially expressed on iso-acceptor level (Fig. 1C). By employing random forest and principal component analysis for feature selection in 37 differentially expressed tsRNA datasets, six tsRNAs were selected for further analyses, namely, tsRNA-Leu-CAG, tsRNA-Pro-CGG, tsRNA-Gln-CTG, tsRNA-Cys-GCA, tsRNA-Leu-AAG, and tsRNA-Lys-CTT (the mapping information on the single-base resolution of their precursor tRNAs was presented in supplemental Fig. 1A–F and Supplemental Table 3). Subsequently, the expression level of 6 tsRNAs was assessed by RT-qPCR analyses and was applied to an internal validation cohort, which included 100 DLBCL patients. The overall survival data was used for the Cox proportional hazards modeling with a 1000-times repeated 10-fold cross-validation strategy (Fig. 1D). As a result, a risk score (RS) was obtained to assess the risk levels of DLBCL patients based on the weighted coefficients as follows (EXP indicated the expression level of tsRNA):

$$\begin{array}{l}{RS}=-0.5042\times {EXP}\left({{Leu}}^{{CAG}}\right)+0.1995\times {EXP}\left({{Pro}}^{{CGG}}\right)\\\quad\quad\;\;\, +\,0.0279\times {EXP}\left({G\mathrm{ln}}^{{CTG}}\right)+\left(-13.17\right)\times {EXP}\left({{Cys}}^{{GCA}}\right)\\\quad\quad\;\;\, +\,0.4851\times {EXP}\left({{Leu}}^{{AAG}}\right)+0.2397\times {EXP}({{Lys}}^{{CTT}})\end{array}$$

The optimal cut-off of RS was −0.8826 by X-tile plots. Notably, the c-index of 6-tsRNAs models for OS prediction was 0.86. The correlation between RS and the characteristics of patients was summarized in Fig. 1E. We observed that RS was significantly correlated with patients’ histological subtypes and stages (Fig. 1F, G). According to the International prognostic index system (IPI), patients were categorized into low, intermedium, and high-risk groups. Our results also demonstrated that patients with low risk have a lower RS score than the other two groups (Fig. 1H) in both cohorts, suggesting that the 6-tsRNA classifier might be an optimal risk prediction biomarker for DLBCL.

**Fig. 2:**
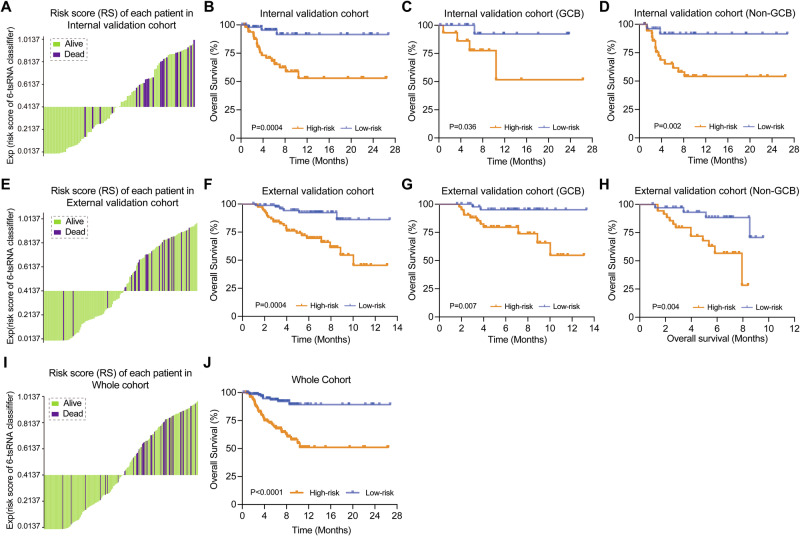
Risk scores of overall survival based on the 6-tsRNA classifiers in DLBCL patients. **A** Risk score and patient survival status are classified by the 6-tsRNA classifiers in the internal validation cohort (*n* = 100). **B** Kaplan–Meier estimated the overall survival (OS) of different risk groups based on 6-tsRNA classifiers’ risk scores in the internal validation cohort (*n* = 100). **C**, **D** Kaplan–Meier estimated the overall survival of different risk groups based on RS in GCB and non-GCB subgroups of the internal validation cohort (*n* = 100). **E** The 6-tsRNA classifiers classified the risk score and DLBCL patient survival status in the external validation cohort (*n* = 160). **F** In the external validation cohort (*n* = 160), Kaplan–Meier estimated the overall survival of different risk groups based on 6-tsRNA classifier risk scores. **G**, **H** Kaplan–Meier estimated the overall survival of different risk groups based on RS in GCB and non-GCB subgroups of the external validation cohort (*n* = 160). **I** The risk score and patient survival status were classified by the 6-tsRNA classifiers in the whole cohort (internal + external validation cohort, *n* = 260). **J** Kaplan–Meier estimated the overall survival of different risk groups based on 6-tsRNA classifier risk scores across the whole cohort (internal + external validation cohort, *n* = 260).

In the internal validation cohort, the Kaplan–Meier analysis showed that patients with higher RS had significantly shorter overall survival (OS) (HR 6.657, 95% CI 2.827–15.68, *P* = 0.0004) (Fig. 2A, B) and progression-free survival (PFS) (HR 4.916, 95% CI 2.273–10.63, *P* = 0.001) (Supplemental Fig. 2A), and the 2-year OS was 91.52% in the lower RS group vs. 52.93% in the higher RS group, the 2-year PFS was 87.04% in the lower RS group vs. 43.29% in the higher RS group. Subgroup analysis also showed that the six tsRNAs classifier had prognosis prediction values in patients with GCB and non-GCB groups (Fig. 2C, D and Supplemental Fig. 2B, C). In the external validation cohort, shorter OS (HR 5.947, 95% CI 2.004–17.66, *P* = 0.0004) and PFS (HR 4.010, 95% CI 2.062–7.798, *P* = 0.0002) in high-risk patients were verified (Fig. 2E, F and Supplemental Fig. 2D), as well as in the patients with GCB and Non-GCB (Fig. 2G, H and Supplemental Fig. 2E, F). In addition, shorter OS (HR 5.374, 95% CI 3.099–9.316, *P* < 0.0001) and PFS (HR 4.247, 95% CI 2.453–7.354, *P* < 0.0001) in high-risk patients was also apparent in the entire cohort of 260 patients (Fig. 2I, J and Supplemental Fig. 2G).

**Fig. 3:**
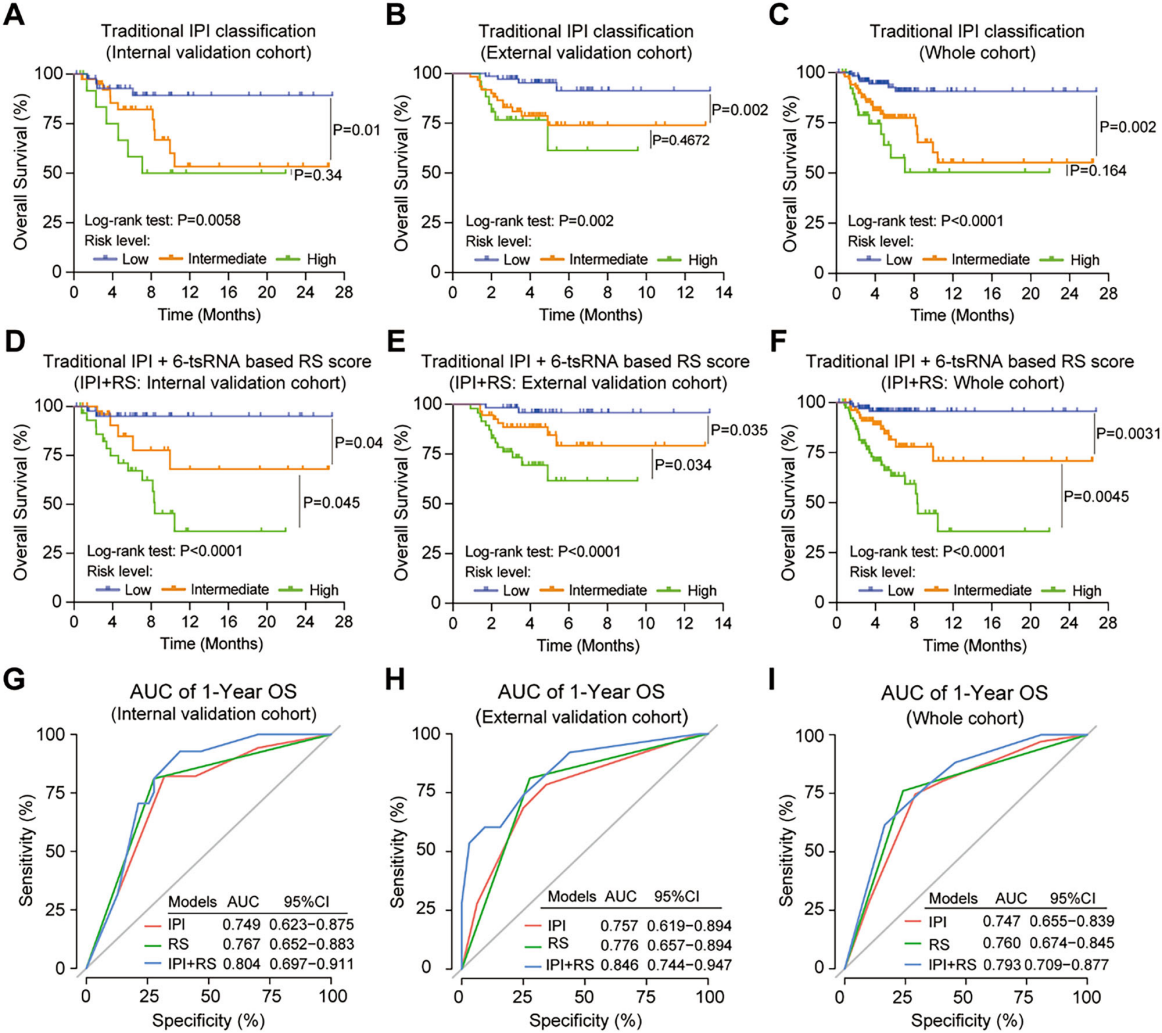
Kaplan–Meier estimated the overall survival of traditional IPI classification and IPI + RS in different cohorts. **A**–**C** Kaplan–Meier estimated the overall survival of traditional IPI classification in the internal validation cohort (*n* = 100), the external validation cohort (*n* = 160), and the whole cohort (internal + external validation cohort, *n* = 260). **D**–**F** Kaplan–Meier estimated the overall survival of IPI + RS classification in the internal validation cohort (*n* = 100), the external validation cohort (*n* = 160), and the whole cohort (the internal + external validation cohort, *n* = 260). **G**–**I** ROC curve of IPI, IPI + RS in the internal validation cohort (*n* = 100), the external validation cohort (*n* = 160) and the whole cohort (internal + external validation cohort, *n* = 260).

Moreover, regarding the IPI scoring system, the Kaplan–Meier analysis showed that the intermediate-risk group had inferior OS than the low-risk group in the internal validation cohort, external validation cohort, and whole cohort, but there was no difference between the intermediate- and high-risk groups (Fig. 3A–C) and PFS (Supplemental Fig. 3A–C). When integrating RS into IPI (Supplemental Table 4), we found that the novel model could discriminate the intermediate-risk group from the low-risk and high-risk groups of different cohorts in OS (Fig. 3D–F). Regarding the discriminate ability in PFS, similar results could also be found in the external validation cohort and the whole cohort but failed in the internal validation cohort (Supplemental Fig. 3D–F). To further evaluate the risk stratification power of IPI + RS in patients, time-dependent ROC curves were plotted, and corresponding AUC was calculated to compare the predictive accuracy of IPI + RS with IPI and RS. The AUCs of OS for IPI + RS were more significant than those of the other prognostic models (Fig. 3G–I and Supplemental Table 5). Multivariable analysis showed that the stage and RS were solely prognostic factors of the overall survival (Supplemental Table 6). Collectively, the results demonstrated that six tsRNAs were a novel promising biomarker for DLBCL malignancy and prognosis prediction.

### Dynamics of 6-tsRNA during therapy effectively evaluate the curative effect

**Fig. 4:**
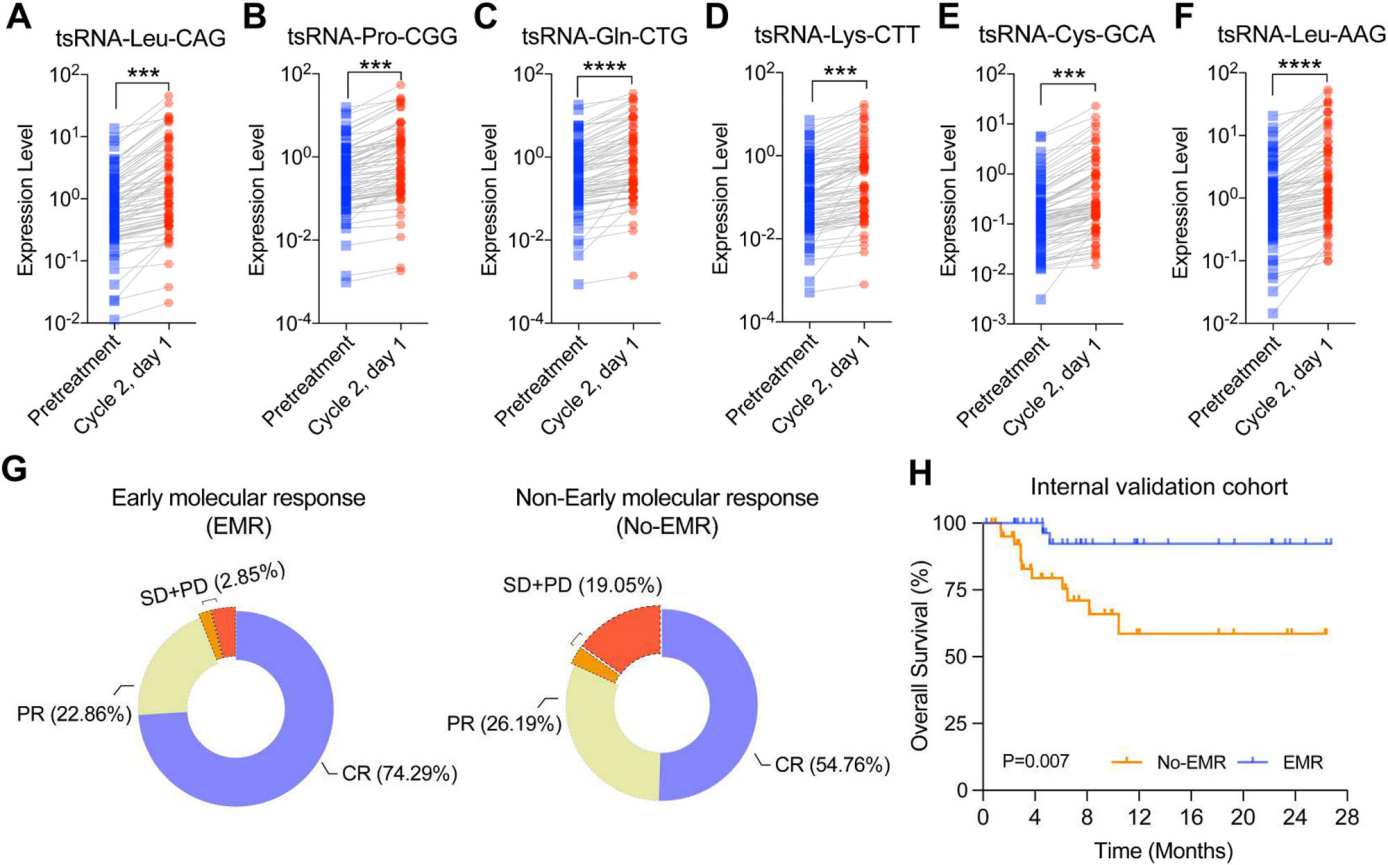
Dynamics of individual tsRNAs and RS during therapy. **A**–**F** The relative expression of tsRNA-Leu-AAG, tsRNA-Gln-CTG, tsRNA-Pro-CGG, tsRNA-Leu-CAG, tsRNA-Lys-CTT, and tsRNA-Cys-GCA at pre-treatment and cycle 2, day 1. **G** Comparison of interim response assessment in DLBCL patients with the early molecular response (EMR) and non-early molecular response (No-EMR) at cycle 2, day 1. **H** Kaplan–Meier estimated the overall survival of different groups based on EMR in the internal validation cohort.

In clinical practice, interim PET/CT assessment was usually conducted to evaluate the treatment response after four cycles of immune-chemotherapy administration, and increasing evidence has shown that interim PET/CT results were a prognostic factor for DLBCL patients. To figure out the disease monitoring value of the 6-tsRNA classifier, we collected 77 paired serum samples (in total 154 samples) at baseline (pre-treatment) and the beginning of the second chemotherapy cycle (cycle 2, day 1) to detect the expression of 6 tsRNAs. As expected, the expression level of the six tsRNAs of day 1 of cycle 2 was significantly higher than that of pre-treatment. (Fig. 4A–F). Combining interim PET/CT results and the RS decline between the two time points, ROC curves determined the cut-off, and we set the value of 1.06-fold change as a critical threshold to evaluate effective response in our cohort (based on the results of X-tiles). Specifically, the RS drop ≥1.06-fold was defined as an early molecular response (EMR); otherwise, it was defined as a non-early molecular response (No-EMR). Notably, we found that the complete remission (CR) fraction was higher in patients with EMR at interim PET/CT assessment compared with patients with No-EMR (CR: 74.29% vs. 54.76%), and the overall response fraction was also higher in patients with EMR (CR + PR: 97.15% vs. 80.95%) (Fig. 4G). Moreover, the Kaplan–Meier analysis showed that patients who achieved EMR had significantly longer survival (HR 6.10, 95% CI: 2.054–18.12, *P* = 0.007) (Fig. 4H and Supplemental Fig. 4A).

Taken together, these results suggest that dynamic RS assessment could reflect the disease status and predict therapy response in advance. Patients failing to show EMR after the first cycle might be unsuitable for traditional therapy, and a more intensive strategy was needed in the early treatment process.

### Dysregulation of 6-tsRNA signatures in the serum of DLBCL patients

**Fig. 5:**
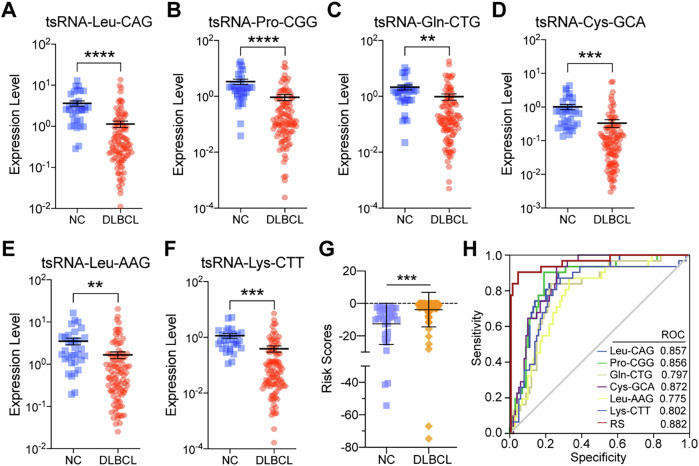
Differential expression analysis of serum tsRNAs in DLBCL patients. **A**–**F** The relative expression of tsRNA-Leu-AAG, tsRNA-Gln-CTG, tsRNA-Pro-CGG, tsRNA-Leu-CAG, tsRNA-Lys-CTT, and tsRNA-Cys-GCA between patients and healthy individuals. **G** Comparison of RS between newly diagnosed DLBCL patients and healthy individuals. **H** ROC curves of individual tsRNAs and RS in serum samples from DLBCL patients versus healthy individuals.

We also compared the expression level of 6 tsRNA classifiers among healthy individuals and DLBCL patients. As the results showed, the expression of tsRNA-Leu-AAG, tsRNA-Gln-CTG, tsRNA-Pro-CGG, tsRNA-Leu-CAG, tsRNA-Lys-CTT, tsRNA-Cys-GCA in healthy individuals was significantly higher than that of DLBCL patients (Fig. 5A–F). Interestingly, we also found that the RS of healthy control was lower than that of patients (Fig. 5G); then, we evaluated the effects of the classifiers on disease diagnosis. ROC curve analysis demonstrated that the six tsRNAs have higher AUC, and the RS has more diagnostic value with AUC 0.882 (95% CI: 0.826–0.939) (Fig. 5H).

Collectively, these data revealed that 6-tsRNAs classifiers might be potential diagnostic biomarkers for DLBCL. (Note that in our study above, we first validated the dysregulation of 6-tsRNA signatures in the serum in the training set (including 100 patients and 35 healthy individuals, as the internal validation set) and then the left-out samples (as the external validation set) were used in the testing set—at this stage, we only used the 100 DLBCL patients) to evaluate the prediction accuracy of tsRNA models) (Refer to Methods for details).

## Discussion

In this study, we systemically identified and displayed a footprint of sncRNA expression profiles of DLBCL for the first time and selected a 6-tsRNA signature as a novel promising biomarker for monitoring DLBCL treatment and prognosis prediction. The 6-tsRNA signatures were significantly correlated with the tumor stage, the predictive risk scores, and the disease categories and had a promising prognosis prediction value applied in clinical treatment responses. Moreover, our work demonstrated that patients who failed to achieve EMR had a lower CR fraction and poor clinical outcomes; thus, such patients might need intensifying therapy in the early stage. Nevertheless, our research identifies novel diagnostic, treatment response, and therapeutic tsRNA targets for DLBCL, supported by relevant literature and data from other cancer types, including solid tumors and treatment-driven biomarkers.

Immunochemotherapy employing rituximab, cyclophosphamide, doxorubicin, vincristine, and prednisone (R-CHOP) is the standard therapeutic regimen for patients with CD20 (+) DLBCL (diffuse large B-cell lymphoma). Nonetheless, a considerable proportion, approximately 30–40%, of patients continue to manifest relapsed or refractory disease and show a poor response rate to salvage therapy [43]. Current treatment response criteria as end-of-treatment for non-Hodgkin lymphoma patients treated with R-CHOP rely on CT scans or F-fluoro-2-deoxy-d-glucose positron emission tomography (FDG-PET) [44]. Still, the imaging method only provides macro overviews of tumor volume and location, which can’t monitor dynamic tumor response to treatment on spatiotemporal single-cell clonal evolution [45] and dominant clonal switching [15]. Such a gap exists in knowledge.

Liquid biopsy is an effective and non-invasive method that detects the molecular characteristics of tumors in peripheral blood and other fluids, such as ascites, effusions, and cerebrospinal fluid, which is much more convenient than tissue biopsy for assessing the heterogeneity and real-time monitoring of the lymphoma. Recently, research of cell-free DNA/circulating tumor DNA (cfDNA/ctDNA), circulating tumor cells (CTCs), extracellular vesicles, proteins, and cell-free ribonucleic acids have made significant progress. Among the various components in a liquid sample, ctDNA can be distinguished from normal cfDNA fragments based on primary tumor-specific genetic or epigenetic alterations. Due to this distinct characteristic, ctDNA has been the most widely investigated marker for cancer detection, treatment monitoring, and identifying residual disease or potential relapse risk. Our previous work, along with other studies [46–48] has demonstrated the feasibility of ctDNA assessment in hematological malignancies. However, ctDNA-based strategies have certain limitations, which our selected 6-tsRNA signature may overcome. Thus, our signature offers a novel and promising biomarker for monitoring DLBCL treatment and predicting prognosis.

First, the detection capability required improvement for early-stage cancer, indolent lymphoma, or low tumor burden is often beyond current techniques. Second, ctDNA assessment can be confounded by clonal hematopoiesis of indeterminate potential, especially in elderly patients. In addition, Cancer Personalized Profiling by Deep Sequencing (CAPP-seq) is a widely utilized sequencing method of ctDNA, which requires detailed knowledge of the underlying genetic landscape of the tumor, and the sequencing panel from different centers varied greatly. If this issue could be solved quickly, the utility of such methods in lymphoma would guide the design of the personal treatment strategy. The tsRNA is another important novel component of the “Liquid biopsy” biomarker. Recently, evidence has shown that tsRNAs are not randomly degraded fragments of tRNA but are a cluster of conserved RNA with stable structures [49, 50] that exert functional regulatory factors in physiological processes and cellular metabolisms [38, 51]. tsRNA species could interact with proteins or mRNA, regulate gene expression, control the cell cycle, and regulate chromatin and epigenetic modifications-related signal transduction pathways [52].

Third, serum detection alone may not provide detailed molecular and cellular context to understand the tumor environment. In contrast, tissue analysis, such as that obtained from lymph node biopsies, provides a more comprehensive understanding of the tumor itself. By directly assessing the expression of tsRNA in the microenvironment of malignant B cells, tissue samples provide valuable information about the biological processes occurring within the tumor. Similar to histology and immunohistochemistry (IHC) in the diagnosis of DLBCL, this method can uncover significant molecular interactions and genetic abnormalities that are essential for a precise diagnosis as well as individualized treatment plans. Integrating serum and tissue evaluations reflects the complementary role each plays in the treatment-response tracking of DLBCL.

In our study, we proposed a non-invasive, cost-effective approach for diagnosing and predicting the prognosis of DLBCL, a type of liquid cancer. We plan to focus on tsRNA modulation in solid tumor tissue to explore the mechanisms behind tsRNA deregulation and potential strategies to reverse it. A relevant approach surfaced in colorectal cancer. tRF/miR-1280, a 17-bp fragment derived from tRNA-Leu in colorectal cancer and pre-miRNA, could directly target Notch ligand JAG2 with the functional consequence of inactivation of Notch signaling suppressed CSC phenotypes by direct transcriptional repression of the Gata1/3 and miR-200b genes constantly elevated levels of JAG2, Gata1, Gata3, Zeb1, and Suz12 [53]. 5′-tRF-Glu-CTC inhibits cell proliferation in high-grade serous ovarian cancer (HGSOC) by targeting the BCAR3 3′-UTR. tRF5-Glu binds directly to a site in the 3′UTR of the breast cancer anti-estrogen resistance 3 (BCAR3) mRNA, downregulating its expression and inhibiting ovarian cancer cell proliferation [54].

Fourth, functionally, the 5′-tRF derived from tRNA-Gly (tRF-03357) downregulates the levels of HMBOX1, thereby promoting cell proliferation, migration, and invasion in HGSOC in vitro [55]. In addition, a novel family of tRFs formed from tRNA(Glu), tRNA(Asp), tRNA(Gly), and tRNA(Tyr) displace the RNA-binding protein YBX1 from several carcinogenic transcripts in breast cancer cells [29]. These fragments have a pattern that matches the YBX1 recognition sequence, making the post-transcriptional silencing sequence specific. These studies highlight the crucial role tsRNA plays in carcinogenesis and cancer progression, making their results particularly relevant to our investigation and strengthening the case for tsRNA as a possible cancer biomarker.

Fifth, Dysregulated expression of tsRNA has been reported in some studies of solid tumor tissues. In a study of gastric cancer, the expression levels of tiRNA-5034-GluTTC-2 in paired gastric cancer tissues and adjacent normal tissues, plasmas from patients with gastric cancer, and healthy people were compared. They found that tiRNA-5034-GluTTC-2 was down-regulated in tumor tissue and plasmas, and these patients with the lower expression exhibited poor outcomes [56]. Another study from chronic lymphocytic leukemia and lung cancer showed that ts-3676, ts-4521, ts-46, ts-47, ts-49, ts-53, and ts-101 were down-regulated and mutated, which can form complexes by interacting with Piwi-like protein 2 (PIWIL2) [57]. Apart from these, several tsRNAs were found to be dysregulated in breast cancer [58]., lung cancer [59, 60], Burkitt lymphoma [61], hepatocellular carcinoma [62], papillary thyroid cancer [63], pancreatic cancer [64], and ovarian cancer [65]. All these studies suggested that tsRNAs could act oncogenic or tumor-suppressor function in cancer. In our study, we found that tsRNAs were lowly expressed in DLBCL patients compared with healthy individuals, which might be caused by tissue specificity, and a further mechanism was needed to figure it out. Furthermore, we created a 6-tsRNA signature in DLBCL patients and found that the novel classifier was correlated with tumor burden, prognosis, and treatment response, suggesting that the assessment of tsRNA in DLBCL was feasible. Thus, combined with the convenience of detection methods, we can conclude that the evaluation of tsRNA might be optimal for individual prognosis prediction and treatment decision-making.

Sixth, early identification of non-responders to initial treatment was necessary for the improvement of the patient’s outcome, which could provide an opportunity for early intervention with appropriate treatment regimens, such as new targeted therapies, autologous bone marrow transplantation, or chimeric antigen receptor T cells [66, 67] Limited to low specificity and sensitivity of interim PET-CT, alternative methods are urgently needed. Some studies showed that molecular response (significant molecular response (MMR): 2.5-log drop in ctDNA after one cycle; early molecular response (EMR): 2-log drop after two cycles) was an independent response predictive biomarker for the DLBCL patients treated with front-line therapy or salvage therapy [46]. Li et al. also found that the dynamics of ctDNA after two cycles effectively predicted response and survival in a Chinese DLBCL cohort (Clinical implications of circulating tumor DNA in predicting the outcome of diffuse large B cell lymphoma patients receiving first-line therapy). Our present study found that pre-treatment 6-tsRNA scores and score dynamics as early as 21 days in therapy were prognostic for a therapeutic window [68]. The tsRNA expression was upregulated during the therapy, and optimal thresholds for the change in 6-tsRNA scores were 1.06 drops (EMR: early molecular response). Patients who achieved the early response have a higher interim CR/PR fraction [the fraction of complete response (CR) and partial responses (PR)] and better outcomes, suggesting that molecular response defined by the 6-tsRNA classifier is potentially applicable in patients with DLBCL. In our study, the patients who did not achieve their favorite EMR scores showed poor therapy response, and such patients might need intensifying therapy; however, to expand the clinically applicable value, future trials should include more patients and long-term follow-up.

Lastly, our present study created a 6-tsRNA signature in DLBCL patients using the deep learning model (1000 times repeated 10-fold cross-validation). As the formula displayed, we found DLBCL [1] patients at diagnosis have lower tsRNA expression, and [2] patients who get CR upon treatment usually show an increase in the expression of the 6 tsRNAs, and [3] bringing these EXP(tsRNA) into the RS equation usually gets a negative RS score [4] lower RS tsRNA score represents high tsRNA levels and implies restoration to healthy levels [5]. we found that the weighted power of tsRNA-Cys-CAG was extremely high. Then, prognosis prediction significance was also checked utilizing the same model, revealing that the expression of tsRNA-Cys-CAG could predict the prognosis significantly. Still, the C-index for OS prediction was 0.73. Generally, all the above suggested that tsRNA-Cys-CAG might play a crucial role in lymphomagenesis and treatment response. Still, the efficacy of prognosis prediction was not as precise as the 6-tsRNA classifier. In addition, we also tested the prognosis prediction value of other combinations, finding that the 6-tsRNA classifiers have more prognosis prediction precision power than other combinations (specifically as follows, when abandoning tsRNA-Leu-CAG, the C-index of the remaining five-tsRNA models was 0.663; when abandoning tsRNA-Pro-CGG, the C-index was 0.662; when abandoning tsRNA-Gln-CTG, the C-index was 0.673; when abandoning tsRNA- Leu-AAG, the C-index was 0.661; and when abandoning tsRNA-Cys-CAG, the C-index was 0.724.). Thus, future experiments with multiple biomarkers could gravitate toward subclone-specific therapeutic targeting and suppressing subclonal evolutionary changes that evolve disease stages and treatment options [15].

In conclusion, we discovered a 6-tsRNA-based signature as a biomarker for DLBCL therapy monitoring (treatment response) and prognosis. These 6-tsRNA fingerprints strongly correlated with tumor stage, prognostic risk scores, and disease categories and might predict clinical outcomes. Our data also showed that patients who did not reach complete remission (CR) had lower CR rates and worse clinical outcomes, indicating an early increased therapy. Currently, we are working on a large-scale cohort study that includes additional health controls and patients with long-term follow-ups. We hope that our ongoing research will further elucidate the diagnostic potential.

## Supplementary information

Supplemental Methods,Tables, and Figures
