# Supplementary material for: A 6-tsRNA signature for early detection, treatment response monitoring, and prognosis prediction in diffuse large B cell lymphoma
Source: Blood Cancer J. 2025 Apr 28;15(1):79. doi: 10.1038/s41408-025-01267-z (PMC12037784; doi:10.1038/s41408-025-01267-z)
Supplement: Supplementary file 1 — Supplemental Methods,Tables, and Figures [file 41408_2025_1267_MOESM1_ESM.docx]

**Supplemental Tables**

**Supplemental Table 1. Primer sequence of tsRNA**

| tsRNA | | Sequence (5’-3’) |
| --- | --- | --- |
| 3'universal primer | | GCGAGCACAGAATTAATACGACTC |
| tsRNA-Leu-CAG | 5' primer | GTCAGGATGGCCGAGCGGTC |
| tsRNA-Pro-CGG |  | GATTCTCGCTTCGGGTGCGAG |
| tsRNA-Lys-CTT |  | GGGACTCTTAATCCCAGGGTC |
| tsRNA-Leu-AAG |  | GGTAGCGTGGCCGAGCGGTC |
| tsRNA-Cys-GCA |  | GGGGGCATAGCTCAGTGGTAG |
| tsRNA-Gln-CTG |  | GGACTCTGAATCCAGCGATC |
| U6 | 3' primer | CTCGCTTCGGCAGCACATATACT |
|  | 5' primer | ACGCTTCACGAATTTGCGTGTC |

| **Supplemental Table 2. tsRNA sequences** | |
| --- | --- |
| tsRNA sequences (5'-3'3') | |
| tsRNA-Leu-CAG | GUCAGGAUGGCCGAGCGGUC |
| tsRNA-Pro-CGG | GAUUCUCGCUUCGGGUGCGAG |
| tsRNA-Lys-CTT | GGGACUCUUAAUCCCAGGGUC |
| tsRNA-Leu-AAG | GGUAGCGUGGCCGAGCGGUC |
| tsRNA-Cys-GCA | GGGGGCAUAGCUCAGUGGUAG |
| tsRNA-Gln-CTG | GGACUCUGAAUCCAGCGAUC |

**Supplemental Table 3. The variables and definitions of different models**

| **Model and definition (total point)** | **Variable** | **Score** |
| --- | --- | --- |
| **IPI** |  |  |
| Low (0-1) | Age (>60 years vs. ≤60 years) | 1 |
| Intermediate (2-3) | Ann Arbor stage (III-IV vs. I-II) | 1 |
| High (4-5) | ECOG score (≥2 vs. 0-1) | 1 |
|  | Elevated LDH (yes vs. no) | 1 |
|  | Extranodal sites (2 vs. 0-1) | 1 |
| **IPI+RS** |  |  |
| Low (0-1) | Age (>60 years vs. ≤60 years) | 1 |
| Intermediate (2-3) | Ann Arbor stage (III-IV vs. I-II) | 1 |
| High (4-6) | ECOG score (≥2 vs. 0-1) | 1 |
|  | Elevated LDH (yes vs. no) | 1 |
|  | Extranodal sites (2 vs. 0-1) | 1 |
|  | RS (High vs. Low) | 1 |

*Abbreviations: IPI, International Prognostic Index; ECOG PS, Eastern Cooperative Oncology Group performance status; LDH, lactate dehydrogenase; RS, risk scores.*

**Supplemental Table 4. Comparsion of time-dependent ROC of OS for IPI, RS, and IPI+RS in different cohort**

| **Cohorts** | **Group** | **P value** |
| --- | --- | --- |
| Internal validation cohort | IPI *vs.* IPI+RS | 0.008065 |
|  | RS *vs.* IPI+RS | 0.0346 |
| External validation cohort | IPI *vs.* IPI+RS | 0.01157 |
|  | RS *vs.* IPI+RS | 0.01513 |
| Whole cohort | IPI *vs.* IPI+RS | 0.0002993 |
|  | RS *vs.* IPI+RS | 0.006407 |

**Supplemental Table 5. Univariate and multivariate analyses of the overall survival**

| **Prognostic variables** | **Univariate analysis** | | **Multivariate analysis** | |
| --- | --- | --- | --- | --- |
|  | **HR (95% CI)** | ***P value*** | **HR (95% CI)** | ***P value*** |
| Gender (female *vs*. male) | 1.576 (0.662-3.754) | 0.304 |  |  |
| Stage (III-IV vs. I-II) | 2.522 (1.341-4.742) | 0.004 | 2.207 (1.101-4.423) | 0.026 |
| IPI | 1.503 (1.073-2.104) | 0.018 | 0.873 (0.556-1.370) | 0.554 |
| Recurrence status (Yes vs. No) | 0.980 (0.287-3.344) | 0.974 |  |  |
| Histological Subgroup (Non-GCB vs. GCB) | 2.119 (0.775-5.790) | 0.143 |  |  |
| Age(>=53 vs. <53y) | 1.989 (0.800-4.946) | 0.139 |  |  |
| 6-tsRNA classifier  (High *vs.* Low risk) | 6.160 (1.814-20.92) | 0.004 | 5.953 (1.292-27.43) | 0.022 |

**Supplemental Figures**

**Supplemental Figure 1**


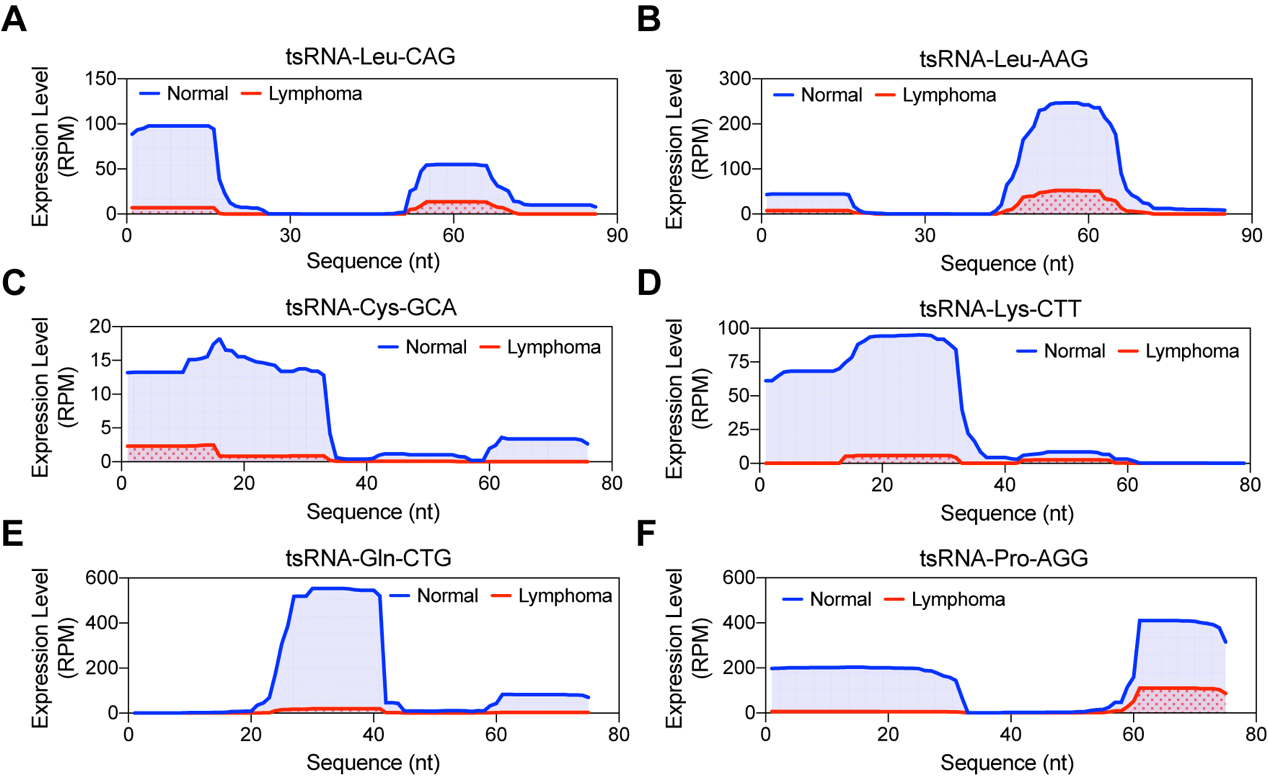


**Supplemental Figure 1. 6 tsRNAs mapping information on their precursor tRNAs.** Structure schematic and nucleotide mapping of (A) tsRNA-Leu-CAG, (B) tsRNA-Leu-AAG, (C) tsRNA-Cys-GCA, (D) tsRNA-Lys-CTT, (E) tsRNA-Gln-CTG, and (F) tsRNA-Pro-AGG on their precursor tRNAs, Expression levels are presented as mean ± SEM

**Supplemental Figure 2**


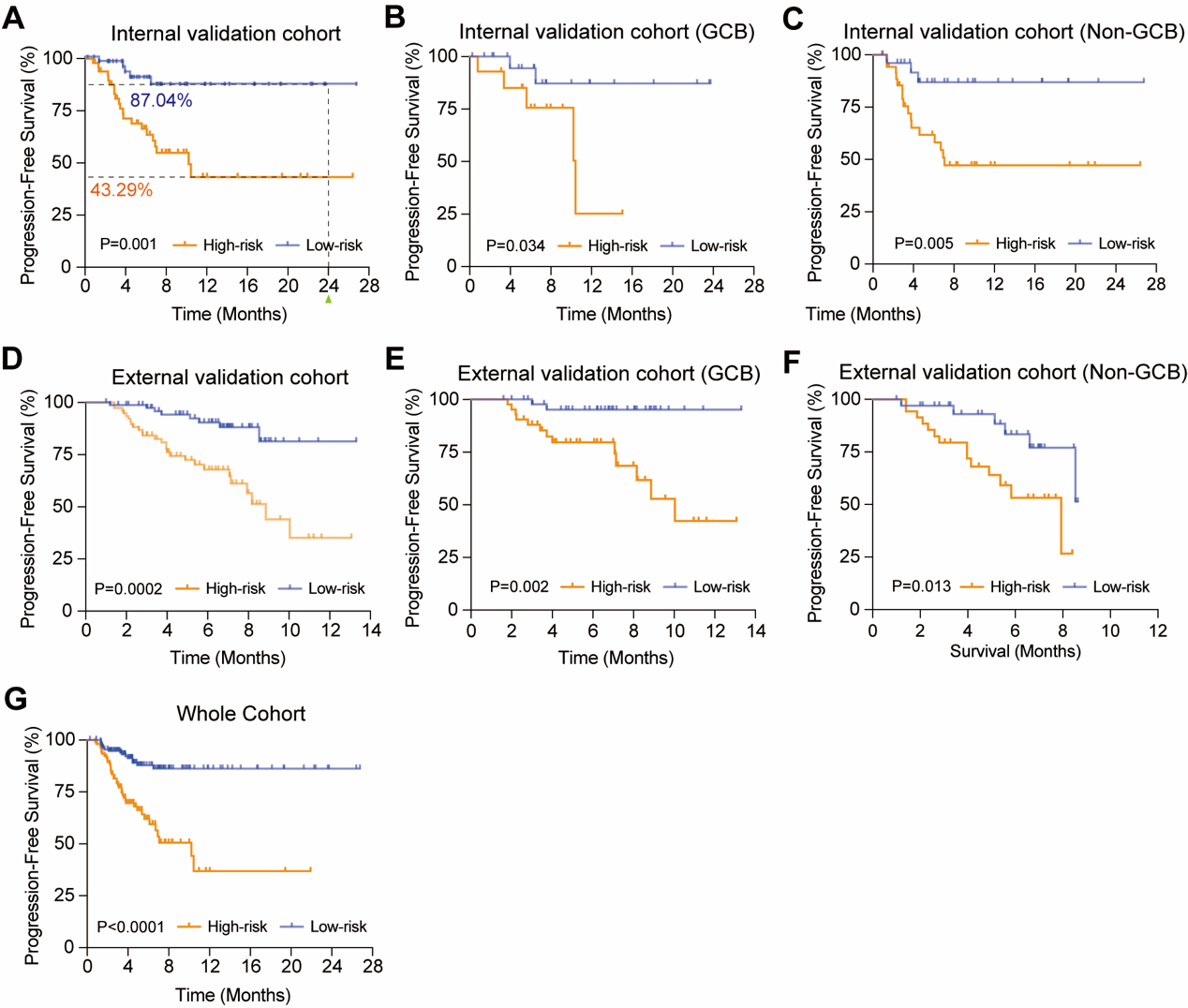


**Supplemental Figure 2. Risk scores of progression-free survival (PFS) based on the 6-tsRNA classifiers in DLBCL patients.** (A) Risk score and patient survival status are classified by the 6-tsRNA classifiers in the internal validation cohort (n=100). (B) Kaplan-Meier estimated the progression-free survival (PFS) of different risk groups based on 6-tsRNA classifiers' risk scores in the internal validation cohort (n=100). (C-D) Kaplan-Meier estimated the progression-free survival (PFS) of different risk groups based on RS in GCB and non-GCB subgroups of the internal validation cohort (n=100). (E) The 6-tsRNA classifier6-tsRNA classifiers classified the risk score and DLBCL patient survival status classified the risk score and DLBCL patient survival status in the external validation cohort (n=160). (F) In the external validation cohort (n=160), Kaplan-Meier estimated the progression-free survival (PFS) of different risk groups based on 6-tsRNA classifier risk scores. (G-H) Kaplan-Meier estimated the progression-free survival (PFS) of different risk groups based on RS in GCB and non-GCB subgroups of the external validation cohort (n=160). (I) The risk score and patient survival status were classified by the 6-tsRNA classifiers in the whole cohort (internal + external validation cohort, n=260). (J) Kaplan-Meier estimated the progression-free survival (PFS) of different risk groups based on 6-tsRNA classifier risk scores across the whole cohort (internal + external validation cohort, n=260).

**Supplemental Figure 3**


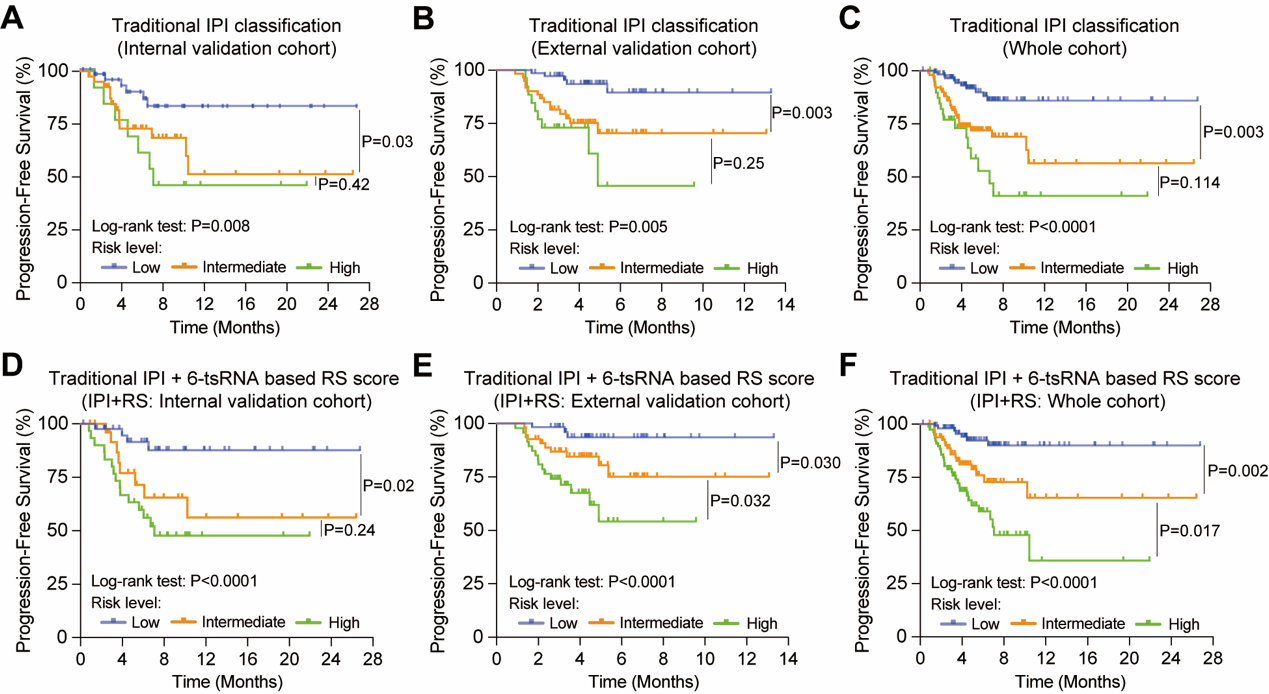


**Supplemental Figure 3. Kaplan-Meier estimated the progression-free survival (PFS) of** **traditional IPI classification and IPI+RS in different cohorts.** (A-C). Kaplan-Meier estimated the progression-free survival (PFS) of traditional IPI classification in the internal validation cohort (n=100), the external validation cohort (n=160), and the whole cohort (internal + external validation cohort, n=260). (D-F). Kaplan-Meier estimated the progression-free survival (PFS) of IPI+RS classification in the internal validation cohort (n=100), the external validation cohort (n=160), and the whole cohort (internal + external validation cohort, n=260).

**Supplemental Figure 4**


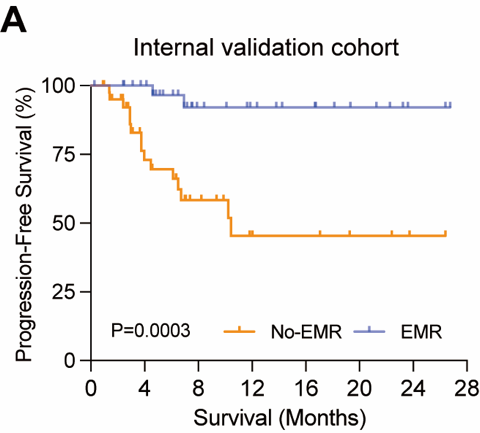


**Supplemental Figure 4. Kaplan-Meier estimated the progression-free survival (PFS) of different groups based on EMR in the internal validation cohort.**
